# Supplementary material for: Exploring prompts to elicit memorization in masked language model-based named entity recognition
Source: PLoS One. 2025 Sep 15;20(9):e0330877. doi: 10.1371/journal.pone.0330877 (PMC12435671; doi:10.1371/journal.pone.0330877)

Appendix

1 MLM-based NER Models

We summarize the model performance details of the 6 explored fine-tuned NER models based on MLMs in Table 1, and more details about the models and accessibility information are described in the following.

Table 1. Self-reported performance of the NER Models on CoNLL-2003 evaluation dataset.

Model	#Param.	P	R	F1	ACC	Download URL
ALBERT-B	11.1M	93.0	93.8	93.4	98.5	`https://huggingface.co/ArBert/albert-base-v2-finetuned-ner`
ALBERT-L	16.6M	94.0	94.5	94.2	98.7	`https://huggingface.co/Gladiator/albert-large-v2_ner_conll2003`
BERT-B	108M	92.1	93.1	92.6	91.2	`https://huggingface.co/dslim/bert-base-NER`
BERT-L	334M	92.0	91.9	92.0	90.3	`https://huggingface.co/dslim/bert-large-NER`
Roberta-B	124M	95.3	96.0	95.7	98.9	`https://huggingface.co/dominiqueblok/roberta-base-finetuned-ner`
Roberta-L	354M	96.2	96.9	96.6	99.4	`https://huggingface.co/Gladiator/roberta-large_ner_conll2003`

ALBERT-B and ALBERT-L NER models are fine-tuned models of base and large versions of ALBERT-v2 respectively. ALBERT-v2 is a transformer-based language model that is pre-trained on a substantial corpus of English text data through self-supervised learning. This pretraining process entails exposure to raw textual data without human annotation, thereby leveraging publicly available data sources. Specifically, ALBERT-v2 utilizes two pretraining objectives: Masked Language Modeling (MLM) and Sentence Ordering Prediction (SOP). Through MLM, the model learns to predict masked tokens within a sequence of text, while SOP tasks involve predicting the correct order of sentences within a document. Notably, ALBERT-v2 aims to optimize efficiency and scalability by parameter sharing across layers and employing parameter reduction techniques, such as factorized embedding parameterization and cross-layer parameter sharing.

BERT-B and BERT-L NER models have the most downloaded records on the public model platform. These two models are fine-tuned models of BERT-base-cased and BERT-large-cased models. BERT is a transformer-based language model renowned for its ability to capture bidirectional contextual information from text data. Similar to ALBERT, BERT models are pre-trained on a large corpus of English data in a self-supervised fashion, aiming to learn deep contextualized representations of words or tokens. BERT employs the MLM objective, where it learns to predict masked tokens within a sequence, and the Next Sentence Prediction (NSP) objective, where it predicts whether two sentences are consecutive in the original text. BERT's architecture comprises transformer encoders stacked on top of each other, allowing it to effectively capture contextual information through attention mechanisms.

RoBERTa-B and RoBERTa-L NER models are fine-tuned models of RoBERTa-base and RoBERTa-large. RoBERTa is another variant of the BERT model designed to improve upon its pretraining methodology and performance. Like BERT and ALBERT-v2, RoBERTa utilizes transformer-based architectures for language modeling tasks. However, RoBERTa introduces several enhancements to the pretraining

process, including dynamic masking strategies, larger training datasets, and longer training times. Notably, RoBERTa replaces the NSP objective with a more extensive masking scheme during pretraining to improve the model's robustness and effectiveness in capturing contextual information from text data. Additionally, RoBERTa employs larger batch sizes and longer training sequences, contributing to its improved performance on downstream natural language processing tasks.

2 Sever Details

All experiments are performed on 1 GPU with 16GB memory of a DGX1 server ((Pascal) Tesla P100).

3 B-PT and W-PT Prompts

Table 2 and 3 shows the best and worst prompts (B-PT and W-PT) for *LOC* and *ORG* entity type respectively.

Table 2. The *LOC* entity *M-MEM* scores and corresponding ranks of the best (Rank=1) and worst (Rank=-1) prompts from the dev set on the test set.

**Model**	**Prompts**	**dev**		**test**	
		**Rank**	***M-MEM***	**Rank**	***M-MEM***
ALBERT-B	What are the most popular tourist spots in MASK?	1	81.19	63	80.24
	Fill up the tank at MASK.	-1	56.73	-1	55.74
ALBERT-L	They founded a tech startup in MASK.	1	82.38	5	81.6
	The university in MASK is highly regarded.	-1	67.4	-1	69.73
BERT-B	Are there any famous landmarks in MASK?	1	73.68	6	72.7
	MASK has the best hiking trails!	-1	66.22	-10	66.61
BERT-L	The national park in MASK is vast.	1	78.39	3	77.6
	Play sports at MASK.	-1	63.32	-3	63.65
RoBERTa-B	MASK is the perfect place for a vacation!	1	78.24	1	80.37
	Tour the campus at MASK.	-1	65.58	-2	67.65
RoBERTa-L	The city hall is in MASK.	1	79.06	1	80.28
	Share your location at MASK.	-1	69.84	-3	68.36

4 Token-level Analysis

We provide token-level analysis for the rest of the five models in Figure 1-5.

5 Self-attention Analysis

We provide self-attention analysis for the rest of the five models in Figure 6.

Table 3. The *ORG* entity *M-MEM* scores and corresponding ranks of the best (Rank=1) and worst (Rank=-1) prompts from the dev set on the test set.

Model	Prompts	dev		test	
		Rank	*M-MEM*	Rank	*M-MEM*
ALBERT-B	Print and display MASK's flyers.	1	76.22	19	76.14
	Contribute an article to MASK's blog.	-1	56.39	-1	58.66
ALBERT-L	What are the main competitors of MASK?	1	75.41	2	77.0
	Listen to MASK's podcast series.	-1	56.75	-1	60.97
BERT-B	The financial performance of MASK has been very impressive.	1	78.46	1	79.63
	Join MASK's virtual workshop.	-1	63.97	-1	67.34
BERT-L	What are the major achievements of MASK?	1	79.1	3	79.94
	Collect signatures for MASK's campaign.	-1	65.9	-1	68.14
RoBERTa-B	What is the mission statement of MASK?	1	77.08	3	76.51
	Submit your artwork to MASK's gallery.	-1	56.82	-1	57.59
RoBERTa-L	What are the main competitors of MASK?	1	74.38	1	75.52
	Collect signatures for MASK's campaign.	-1	58.47	-1	60.47

Fig 1. Analysis of the token importance in prompts. *M-MEM* scores for the **ALBERT-B model**. The best-performing (on the left) and the worst-performing (on the right) prompts were selected on the dev set.

Prompt	M-MEM
Bravo , MASK , what an impressive performance !	74.84
Bravo , MASK , what an impressive performance	75.14
Bravo , MASK , what impressive performance	75.02
Bravo , MASK , what impressive	74.64
Bravo , MASK , impressive	73.29
Bravo MASK , impressive	72.92
MASK , impressive	72.61
MASK impressive	73.03

Prompt	M-MEM
Are you going to MASK 's art gallery opening tonight ?	58.92
Are you going to MASK art gallery opening tonight ?	49.07
Are going to MASK art gallery opening tonight ?	46.37
Are going to MASK art gallery opening tonight	44.58
going to MASK art gallery opening tonight	42.19
to MASK art gallery opening tonight	41.92
to MASK art gallery tonight	42.90
to MASK art gallery	43.29
to MASK gallery	51.44
MASK gallery	60.55

6 Results for *LOC* and *ORG* Entities

6.1 Correlation Analysis

Figures 7-9 show the correlation analysis for *LOC* and *ORG* entities.

6.2 Sentence-level Analysis

Figure 10 and 11 show the sentence-level analysis for *LOC* and *ORG* entities.

Fig 2. Analysis of the token importance in prompts. *M-MEM* scores for the **ALBERT-L model**. The best-performing (on the left) and the worst-performing (on the right) prompts were selected on the dev set.

Prompt	M-MEM	Prompt	M-MEM
Oh , MASK , you 're a true gem in our team .	73.12	MASK , practice forgiveness towards yourself and others .	64.60
Oh , MASK , you 're a gem in our team .	73.14	MASK , forgiveness towards yourself and others .	64.78
Oh , MASK , you 're a gem in team .	73.03	MASK , towards yourself and others .	64.30
Oh , MASK , you 're a gem in team	71.88	MASK towards yourself and others .	63.76
Oh , MASK , you 're a gem team	72.27	MASK towards yourself and .	66.02
Oh MASK , you 're a gem team	72.30	MASK towards yourself .	65.98
Oh MASK , you a gem team	72.32	MASK towards yourself	63.86
Oh MASK , you a team	71.65	MASK towards	68.21
Oh MASK you a team	70.93		
Oh MASK you team	70.41		
Oh MASK team	69.68		
Oh MASK	69.92		

Fig 3. Analysis of the token importance in prompts. *M-MEM* scores for the **BERT-L model**. The best-performing (on the left) and the worst-performing (on the right) prompts were selected on the dev set.

Prompt	M-MEM	Prompt	M-MEM
What project is MASK working on ?	71.56	I had a chance to meet MASK 's family .	67.78
What project is MASK working on	71.78	I had a chance to MASK 's family .	53.09
What project is MASK working	72.33	I had a chance to MASK family .	50.28
What project is MASK	73.03	I had a chance to MASK family	48.68
What is MASK	72.87	I had chance to MASK family	49.56
What MASK	70.50	had chance to MASK family	56.82
		had to MASK family	70.37
		had to MASK	70.17
		to MASK	70.50

Fig 4. Analysis of the token importance in prompts. *M-MEM* scores for the **RoBERTa-B model**. The best-performing (on the left) and the worst-performing (on the right) prompts were selected on the dev set.

Prompt	M-MEM	Prompt	M-MEM
MASK , can you recommend a good restaurant in town ?	70.76	I had a great conversation with MASK at the party .	57.00
MASK can you recommend a good restaurant in town ?	70.97	I a great conversation with MASK at the party .	55.63
MASK can you recommend a good in town ?	71.52	I a great conversation with MASK at the party	55.62
MASK can you recommend a good in ?	72.17	I a conversation with MASK at the party	55.60
MASK can you recommend good in ?	72.68	I conversation with MASK at the party	55.86
MASK you recommend good in ?	71.64	I with MASK at the party	55.29
MASK you recommend in ?	71.20	I with MASK at party	55.87
MASK you recommend in	70.64	with MASK at party	58.01
MASK you recommend	68.50	with MASK at	60.25
MASK recommend	66.61	with MASK	59.41

Fig 5. Analysis of the token importance in prompts. *M-MEM* scores for the **RoBERTa-L model**. The best-performing (on the left) and the worst-performing (on the right) prompts were selected on the dev set.

Prompt	M-MEM	Prompt	M-MEM
MASK , invest in meaningful relationships .	75.27	MASK , practice playing the guitar .	70.61
MASK , invest in meaningful relationships	76.15	MASK , playing the guitar .	69.58
MASK , invest in relationships	75.86	MASK , the guitar .	69.19
MASK , invest in	76.28	MASK , guitar .	69.15
MASK , invest	75.43	MASK , guitar	70.46
MASK invest	74.61	MASK guitar	72.58

Fig 6. Analysis of attention heatmaps. Attention heatmaps of the best and the worst prompts across 5 models on the dev set averaged over all attention heads and layers. The attention weights corresponding to each prompt's "MASK" token are used and averaged over the in-sample and out-of-sample PER sets separately. (a) ALBERT-B. (b) ALBERT-L. (c) BERT-L. (d) RoBERTa-B. (e) RoBERTa-L.

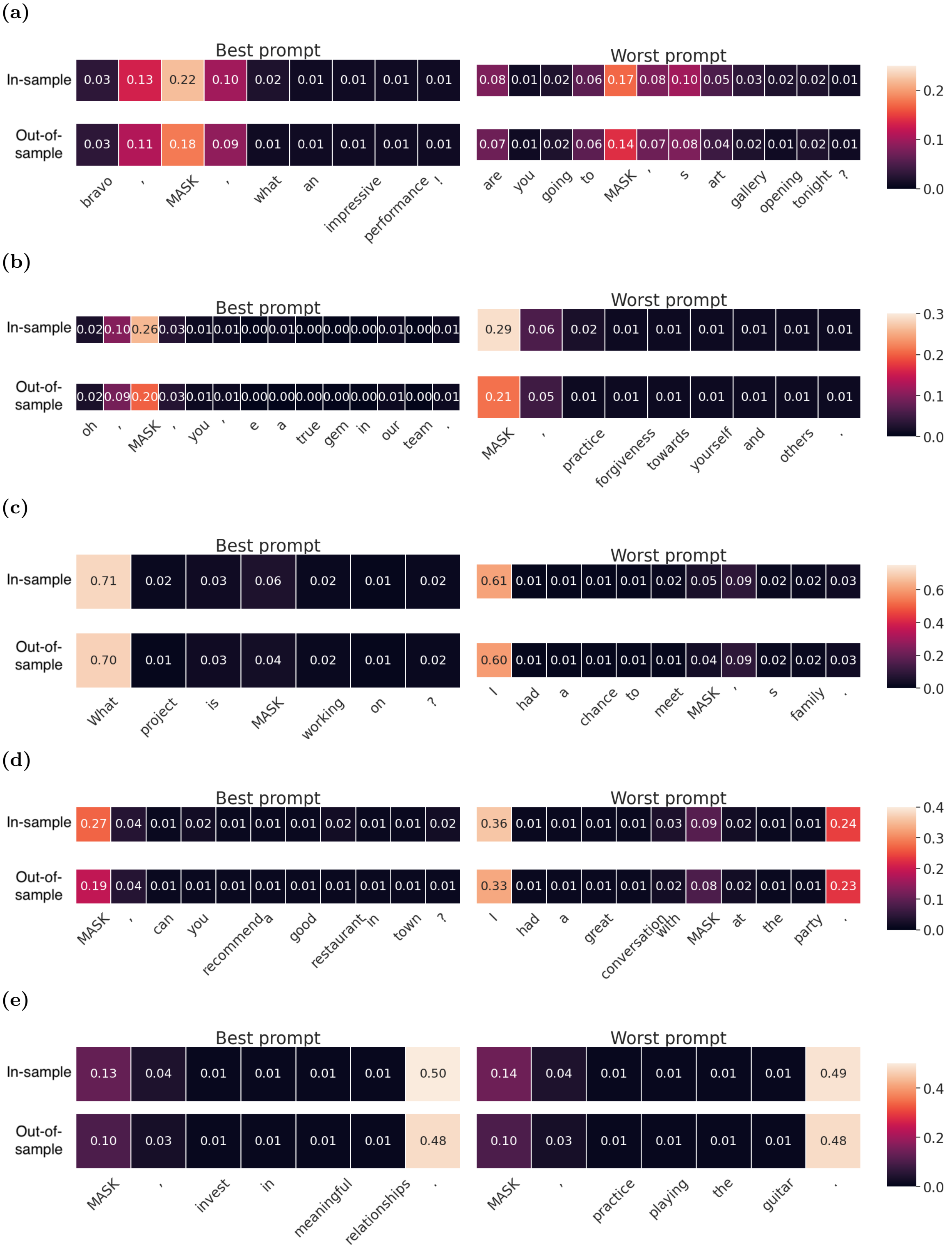

Fig 7. Correlations (Kendall's τ) of the location *M-MEM* scores of prompts across models. Left: correlations for the dev set scores. Middle: correlations for the test set scores. Right: correlations between dev and test set scores.

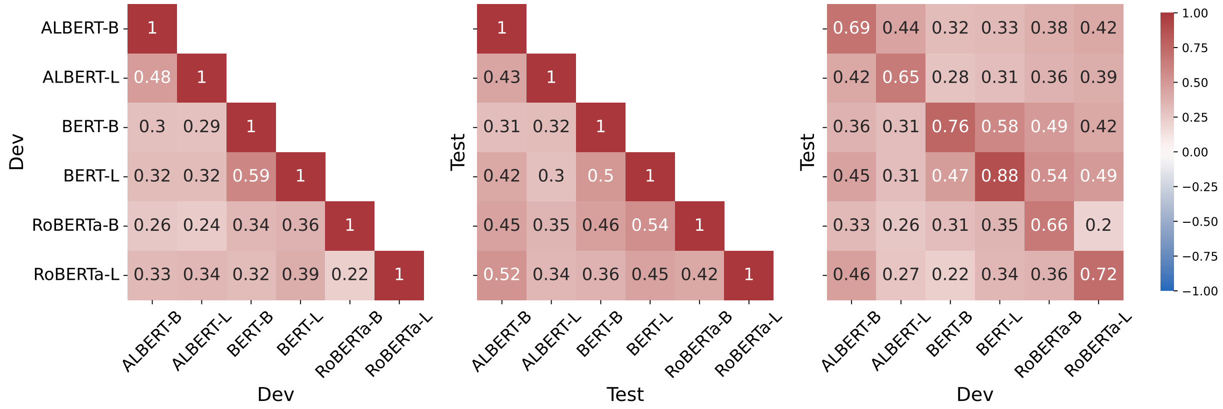

Fig 8. Correlations (Kendall's τ) of the organization *M-MEM* scores of prompts across models. Left: correlations for the dev set scores. Middle: correlations for the test set scores. Right: correlations between dev and test set scores.

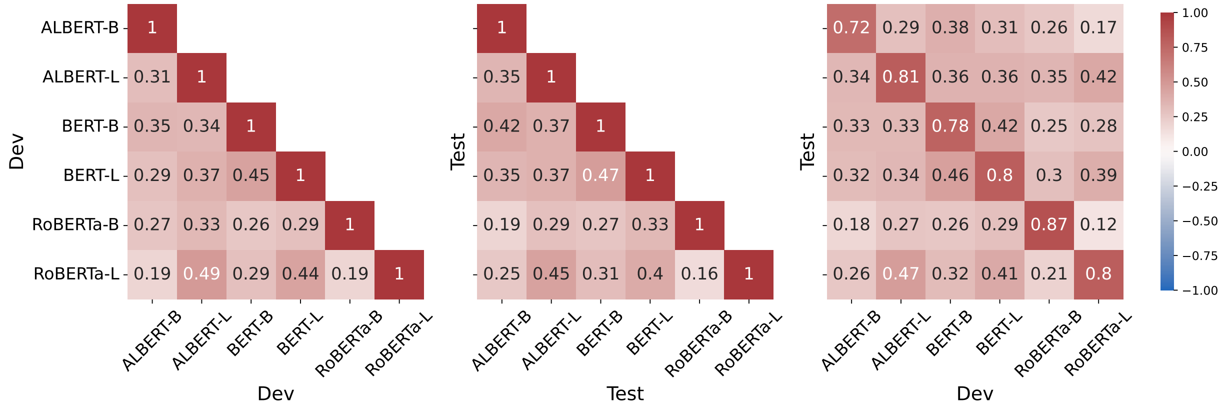

Fig 9. Correlations (Kendall's τ) of the organization *M-MEM* scores of prompts across models. Left: correlations for the dev set scores. Middle: correlations for the test set scores. Right: correlations between dev and test set scores.

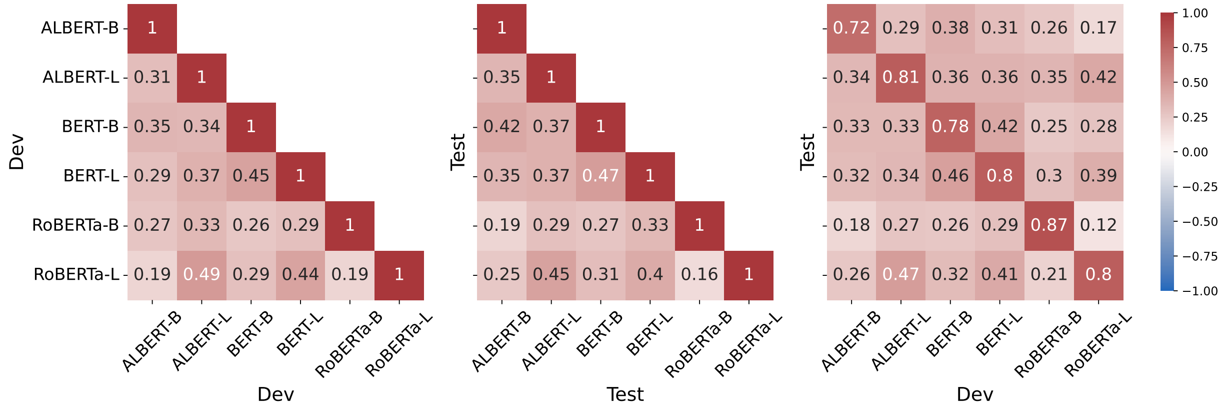

Fig 10. Location *M-MEM* scores grouped by different prompt properties. (a)Prompt types (**dec**larative, **exc**lamatory, **imp**erative, and **int**errogative). (b) Token positions of location entity in the prompt. (c)Prompt token lengths.

(a)

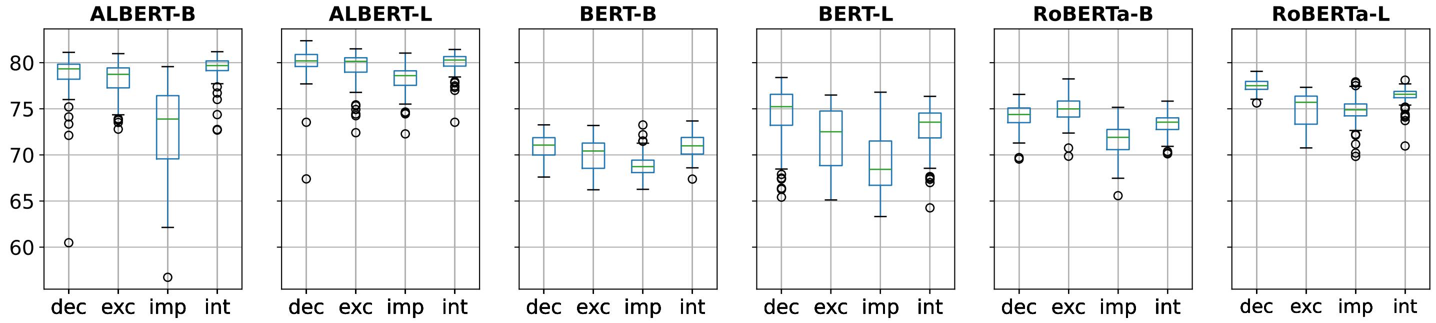

(b)

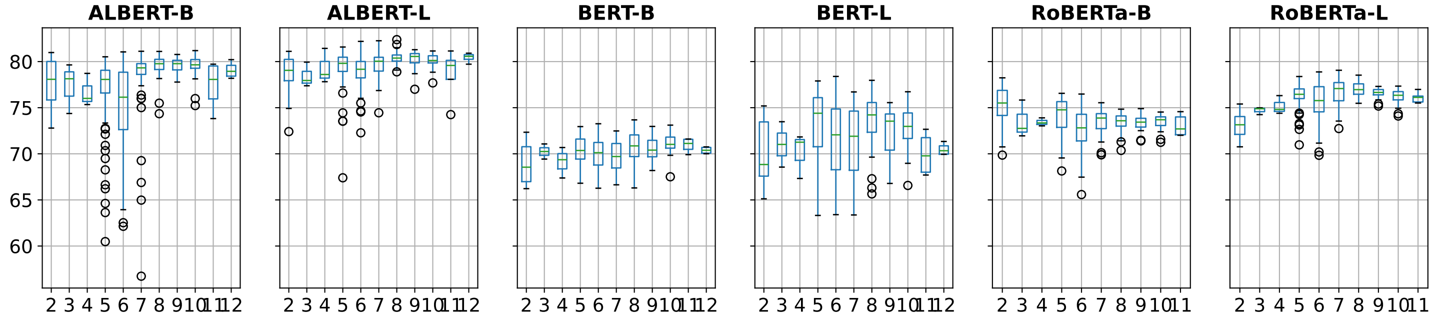

(c)

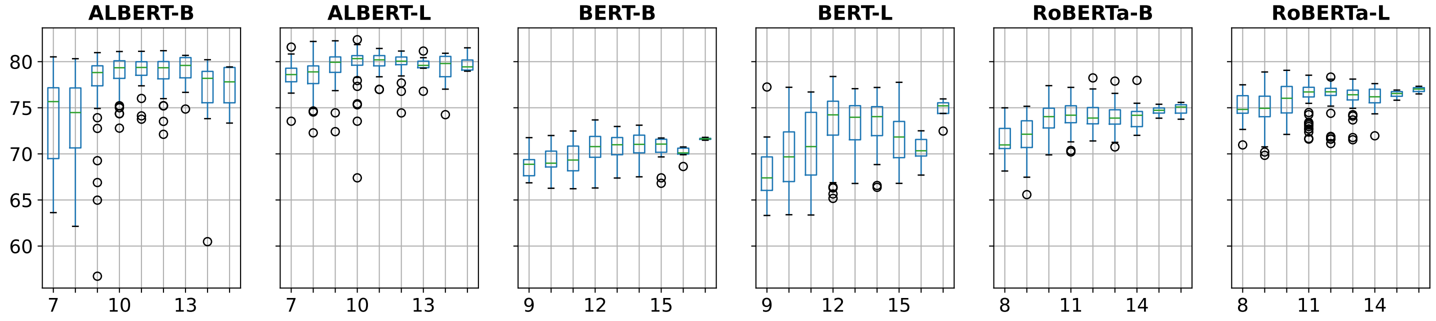

Fig 11. Location *M-MEM* scores grouped by different prompt properties. (a)Prompt types (**dec**larative, **exc**lamatory, **imp**erative, and **int**errogative). (b) Token positions of location entity in the prompt. (c)Prompt token lengths.

(a)

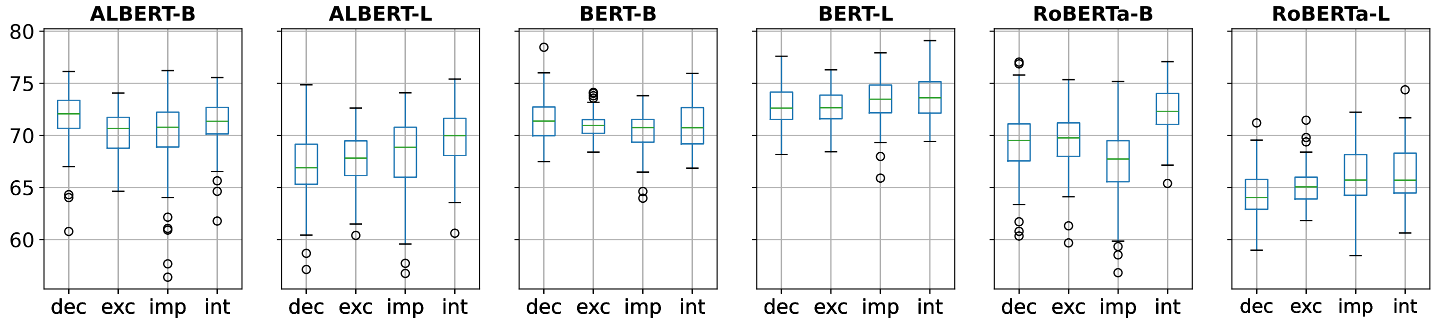

(b)

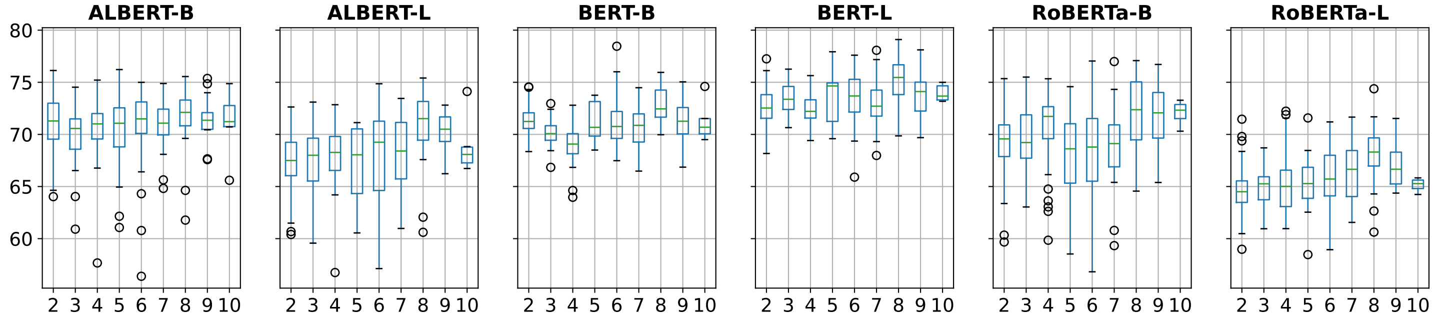

(c)

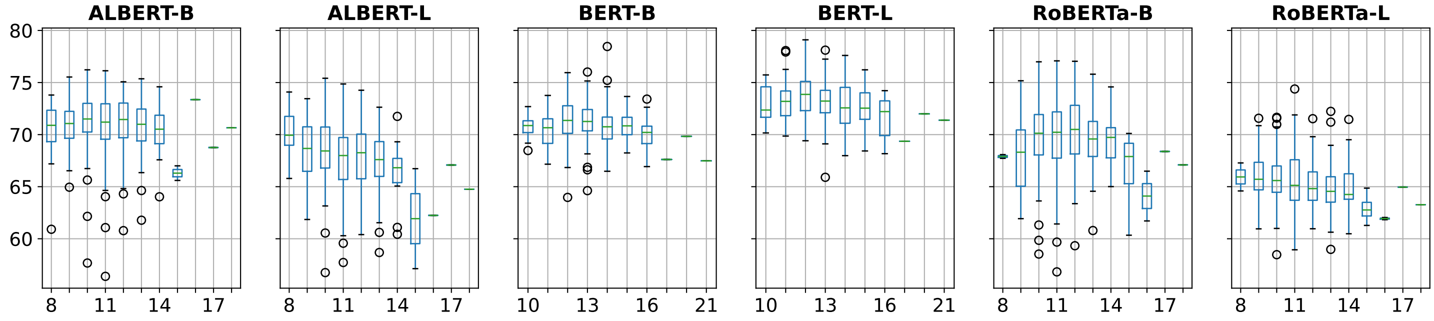

Supplement: S1 Appendix — (PDF) [file pone.0330877.s001.pdf]
