# Supplementary material for: Exploring prompts to elicit memorization in masked language model-based named entity recognition
Source: PLoS One. 2025 Sep 15;20(9):e0330877. doi: 10.1371/journal.pone.0330877 (PMC12435671; doi:10.1371/journal.pone.0330877)
Supplement: Attachment — Submitted filename: Response_to_Reviewers_auresp_2.pdf [file pone.0330877.s004.pdf]

Rebuttal Letter

Dear Editors and Reviewers,

We thank the reviewers for their thoughtful and constructive feedback on our manuscript, titled "Exploring Prompts to Elicit Memorization in Masked Language Model-based Named Entity Recognition" (Manuscript ID: [PONE-D-25-05072]). We greatly appreciate the time and effort dedicated to evaluating our work, and we have carefully considered each comment in preparing our revised manuscript.

Below, we provide a point-by-point response to each reviewer's comment. For clarity, we restate the reviewers' comments in bold, followed by our detailed replies and a summary of the changes made to the manuscript (highlighted in yellow). We believe that the revisions have significantly improved the quality, clarity, and impact of the paper, and we hope the updated version addresses the reviewers' concerns satisfactorily

1. The GitHub link currently points the "main" branch, which is a moving target. Please either create a tag to point to or point to an SHA so the paper will reference a static version of the code.
Response: We have created a tag named v1.0 and updated the GitHub link in the paper to point to a static version of the code.

2a. Reference 12 has subscript "a0" in place of "_a_0" in the DOI.
Response: We have revised "a0" to "_a_0".

2b. Some references use DOIs and others use HTTP links where DOIs are available, and some don't have links despite being available online. I suggest using DOIs where available, and HTTP links where DOI links aren't available but the source is available online. This recommendation is very soft; I don't think this should prevent publication.
Response: We have revised the references by adding necessary DOIs or HTTP links for each referred paper.

Sincerely,
Yuxi Xia
On behalf of all co-authors
